# SLE: Another Autoimmune Disorder Influenced by Microbes and Diet?

**DOI:** 10.3389/fimmu.2015.00608

**Published:** 2015-11-30

**Authors:** Qinghui Mu, Husen Zhang, Xin M. Luo

**Affiliations:** ^1^Department of Biomedical Sciences and Pathobiology, Virginia Tech, Blacksburg, VA, USA; ^2^Department of Civil and Environmental Engineering, Virginia Tech, Blacksburg, VA, USA

**Keywords:** SLE, microbiota, hygiene hypothesis, bacterial antigens, diet, estrogen

## Abstract

Systemic lupus erythematosus (SLE) is a multi-system autoimmune disease. Despite years of study, the etiology of SLE is still unclear. Both genetic and environmental factors have been implicated in the disease mechanisms. In the past decade, a growing body of evidence has indicated an important role of gut microbes in the development of autoimmune diseases, including type 1 diabetes, rheumatoid arthritis, and multiple sclerosis. However, such knowledge on SLE is little, though we have already known that environmental factors can trigger the development of lupus. Several recent studies have suggested that alterations of the gut microbial composition may be correlated with SLE disease manifestations, while the exact roles of either symbiotic or pathogenic microbes in this disease remain to be explored. Elucidation of the roles of gut microbes – as well as the roles of diet that can modulate the composition of gut microbes – in SLE will shed light on how this autoimmune disorder develops, and provide opportunities for improved biomarkers of the disease and the potential to probe new therapies. In this review, we aim to compile the available evidence on the contributions of diet and gut microbes to SLE occurrence and pathogenesis.

## Introduction

The mammalian gut harbors trillions of microorganisms known as the microbiota (1). Increasing evidence in recent years suggest that host microbiota and immune system interact to maintain tissue homeostasis in healthy individuals (2–6). Perturbation of the host microbiota, especially in the gut, has been shown to be associated with many diseases. Among these are autoimmune disorders that include inflammatory bowel disease (IBD) (7, 8), type 1 diabetes (T1D) (9–12), rheumatoid arthritis (5, 13–15), and multiple sclerosis (16, 17). However, little is known on the role of gut microbiota in systemic lupus erythematosus (SLE) (18).

Systemic lupus erythematosus is an autoimmune disorder characterized by severe and persistent inflammation that leads to tissue damage in multiple organs. According to the Lupus Foundation of America, about two million Americans currently live with the disease. The prevalence ranges from 20 to 200 cases per 100,000 persons, with higher prevalence for people of African, Hispanic, or Asian ancestry (19, 20). Although the disease affects both males and females, women of childbearing age are diagnosed nine times more often than men.

Our research team has recently described the dynamics of gut microbiota in a classical SLE mouse model MRL/Mp-*Fas^*lpr*^* (MRL/lpr) (21). In young, female lupus mice, we found marked depletion of *Lactobacilli*, and increase of Clostridial species (*Lachnospiraceae*) together with increased bacterial diversity compared to age-matched healthy controls. Importantly, dietary treatments that improved lupus symptoms in lupus mice also restored gut colonization of *Lactobacillus* and decreased that of *Lachnospiraceae*. In human SLE, a recent cross-sectional study has shown that a lower *Firmicutes* to *Bacteroidetes* ratio was present in women with SLE even after disease remission (22). Similarly, a higher level of *Bacteroidetes* was found in lupus-prone SNF1 mice with more severe disease (23), though this was not evident in MRL/lpr mice (21). These results suggest a potentially important role of gut microbiota on lupus pathogenesis, in particular a potential role of *Bacteroidetes*, since the relative abundance of these bacteria is increased in human SLE and at least one murine lupus model. In this review, we aim to compile the available evidence that associates gut microbes to SLE.

## Environmental Factors and SLE

It is well established that genetic factors influence lupus susceptibility. However, the lack of disease concordance between genetically identical twins strongly suggests the role of non-genetic factors, most likely of environmental factors (24). The role of environmental factors in the etiology of SLE is evidenced by the dramatic difference in disease incidence between West Africans and African Americans, both derived from the same ethnic group but exposed to different environments (25). With an obviously higher burden of infections, the frequency of SLE is much lower in West Africa than Africans living in Europe or USA. The mechanism behind this observation is still unclear, but improvement in hygiene and absence of certain microbes may have contributed to the higher incidence and faster progression of lupus disease (26). In addition to microbes, a number of environmental triggering factors have been described to be associated with SLE, including UV light and cigarette smoking, some of which trigger lupus through epigenetic mechanisms (27–30).

### The Hygiene Hypothesis

Increase of SLE occurrence in the developed world has been reported. Data from several regions of USA show that the incidence of SLE increased at least threefold within the second half of the twentieth century (31, 32). This increase could be related to changes of environmental factors, though better diagnostic methods and increasing awareness of the disease may partially lead to the change in SLE frequency. Similar increase has been observed in a study analyzing the incidence of SLE in Denmark (33). Genome evolution rate seems to be unpersuasive to this increase. In contrast, due to advancements on medicine and vaccination, a number of infectious agents have been gradually eliminated in developed countries, and the sanitation condition has been largely improved. Some have thus proposed that lower exposure to infections leads to the rise of allergies and some autoimmune diseases, such as T1D (34, 35). This is called “The Hygiene Hypothesis.” Considering the rise of SLE frequency in developed countries, it is reasonable to extend the hypothesis to this autoimmune disorder.

Increasing hygiene standards eliminates both pathogenic and non-pathogenic microbes from the environment. Infections from pathogenic microbes, or the lack thereof, are known to be associated with SLE occurrence. Epstein–Barr virus (EBV) and cytomegalovirus (CMV), for example, have been linked to the pathogenesis of SLE by several reports (36–40). Commensal microbes residing inside the host, in return, have been shown to maintain and expand CD8^+^ memory T cells during CMV infection, supporting the notion that microbiota and CMV cooperatively augment immune activation (41). While EBV and CMV are largely considered triggers of SLE, it is increasingly evident that some infections may be beneficial and the lack of them might actually facilitate SLE. In one surprising report (42), two female SLE patients with severe SLE showed improved disease after experiencing infections for a short period of time. Before the infections, both patients failed to respond to a long time of immunosuppressive therapy. Neither experienced relapse after the amelioration of SLE symptoms following the infections. One of the patients even had a successful pregnancy, which is known to trigger lupus flares. Unfortunately, the study did not identify the causing agent that ameliorated the disease. However, another study has identified hepatitis B virus (HBV) as a protective factor against SLE (43). In their study, 2.5% of SLE patients were found positive for the presence of HBV-core antibody, compared to 10.7% from normal controls, which suggests a potential benefit of HBV infection against the occurrence of SLE. In addition, in a large serologic survey, *Helicobacter pylori* seronegativity was found to be associated with an increased risk and earlier onset of SLE in African Americans, suggesting a protective role of *H. pylori* in SLE patients (44, 45). These studies suggest that, in developed countries where HBV and *H. pylori* infections are decreasing (46–48), the risk for developing SLE could become higher. T cell exhaustion during chronic infection may explain the ability of these pathogens to down-regulate inflammation and ameliorate SLE (49, 50).

In lupus-prone mouse models, beneficial roles of some pathogenic microbes have also been suggested. Chen et al. reported that with the infection of *Toxoplasma gondii*, New Zealand Black (NZB) × New Zealand White (NZW) F1 (NZB/W F1) mice had significantly decreased mortality, ameliorated proteinuria level, and reduced anti-DNA IgG in serum. IFNγ and IL-10 expression was reduced in the spleen in the presence of *T. gondii*, suggesting the suppression of T helper 1 (Th1) and Th2 responses, respectively, both demonstrated to be pathogenic in murine lupus (42, 51). In addition, when examining NZB/W F1 mice treated with live *Plasmodium chabaudi*, another prevalent parasite, several independent groups have found that the malaria-causing microbe can prevent clinical symptoms of murine lupus and protect the animals against lupus nephritis (52–54). This is perhaps due to the changed cytokine profile and redox status in both liver and kidney of the mice. Moreover, virus infection has also been found to improve murine lupus symptoms in addition to parasites. For instance, the infection of lactate dehydrogenase elevating virus (LDV) has been shown to significantly suppress the production of anti-nuclear antibody (ANA) and the development of glomerulonephritis in NZB/W F1 mice (26, 55–58). The beneficial effect is hypothesized to be associated with superoxide anion production from macrophages and modulation of prostaglandin E. While LDV and *P. chabaudi* do not infect humans, results from these mouse studies suggest that some infections might be associated with decreased severity of SLE.

### Antibiotics and SLE

Antibiotics, which can remove gut bacteria, are known to trigger lupus flares. These include sulfa drugs such as ­trimethoprim–sulfamethoxazole (Septra), tetracycline-related antibiotics such as minocycline, and penicillin-related antibiotics such as amoxicillin. Increased sun sensitivity with antibiotics may be one mechanism behind the observations. However, antibiotics also cause diarrhea and remove beneficial microbes from the intestinal tract. Could it be the removal of “good” bacteria a mechanism by which antibiotics induce flares in SLE patients? In addition, bacterial metabolites produced by gut microbes can modulate immune function. Recently, several groups have found that metabolites produced by gut bacteria, especially butyrate produced by Clostridia, can promote the differentiation of regulatory T cells (Tregs) in the colon, spleen, and lymph nodes to suppress inflammation (59–62). Thus, removal of certain gut commensals with antibiotics could potentially lead to decreases of bacterial metabolites, such as homoserine lactone, *N*-acetylmuramic acid, and *N*-acetylglucosamine (63) – which could be immunosuppressive – thereby facilitating lupus progression. Incidentally, African Americans have used antibiotics much more frequently than people in West African countries (64, 65), and this may have impacted the differences in lupus prevalence and severity between the two populations.

### Dietary Components and SLE

Diet, one of the main environmental factors with known effects on gut microbiota, has been studied extensively in both SLE patients and lupus-prone mice. Vitamin D (VD), vitamin A (VA), and omega-3 polyunsaturated fatty acids (PUFAs), for instance, have been found to modulate lupus onset or flares. Current knowledge suggests that dietary components can influence SLE through changing the composition and function of gut microbiota, modulating immunological pathways, and/or exerting epigenetic changes (18, 30, 66, 67). Here, we summarize the recent updates on the roles of VD, VA, and PUFAs on lupus.

Vitamin D deficiency is increasingly common, resulting in increased risks for multiple disorders (68, 69). Although VD can be synthesized by the body in sunlight, adequate VD in diet is recommended. VD plays an important role in the homeostasis of the immune system, through a nuclear receptor existing in all immune cells, VD receptor (VDR). Polymorphisms of VDR have been recently reported to be associated with SLE susceptibility (70). In SLE patients, lower VD levels are associated with higher SLE activity. Handono and colleagues found that 1,25(OH)_2_D3 can inhibit neutrophil extracellular trap (NET) formation in cultured cells from SLE patients with hypovitamin D (71). Inhibition of NETs prevents endothelial damage that promotes the progression of lupus disease (72), suggesting a possible benefit of supplying VD in SLE patients with suboptimal VD levels. Recently, it has been reported that VD supplementation increases the number of Treg cells and induces the shift toward Th2 response in pre-menopausal female SLE patients, although a direct efficacy toward disease activity was not observed (73, 74). Likewise, no correlation was found between SLE-associated cytokine profiles and VD levels (75). However, in juvenile-onset SLE, which is more aggressive than adult SLE, dietary intake of VD has been reported to preclude disease progression in several recent studies (75–78). It is worth noting that the doses of VD utilized in these studies were different – one was rather intensive (50,000 international units or IU/week) and the other was more standard (2,000 IU daily) – but the outcomes were similar with improvement of SLE Disease Activity Index. Further studies are required to verify these findings in juvenile-onset SLE, and to explore the mechanisms of why a lack of response to VD was seen in adult SLE.

Vitamin A has long been recognized as an immune regulator. VA exerts its effects mainly via all-*trans*-retinoic acid (tRA), an active metabolite of VA. For SLE, the role of VA has been revealed through oral administration of tRA to either SLE patients or lupus-prone mice. In SLE patients, some benefit of tRA to ameliorate lupus nephritis and proteinuria has been reported (79, 80). For murine lupus, several mouse models, including NZB/W F1 and MRL/lpr, showed reduced proteinuria and renal damage when supplemented with tRA (81–85). In our study (85), although tRA treatment improved lupus-like kidney disease in MRL/lpr mice, there were serious side effects: worsened inflammation in the skin, brain, and lung, as well as increased levels of circulating autoantibodies. Our findings suggest the need to monitor diverse organs in SLE patients if tRA were used as a treatment, avoiding any potential damage to organs other than the kidneys.

Polyunsaturated fatty acids, with the main representative being omega-3 fatty acid, have been studied as complementary or alternative treatments for SLE for many years. Omega-3 PUFAs cannot be synthesized by the human body or other mammals. Eicosapentaenoic acid (EPA) and docosahexaenoic acid (DHA) are two well-recognized members of omega-3 PUFAs that are found in deep sea cold water fish. Fish oil is thereby utilized in some animal studies and clinical trials to test the efficacies of omega-3 PUFAs. In 1980s, DHA and EPA were both demonstrated to ameliorate renal disease, reduce anti-dsDNA autoantibody levels, and prolong lifespan of NZB/W F1 mice (86–88). It was found that fish oil prevents murine lupus by reducing levels of various pro-inflammatory cytokines, including IL-1β, IL-6, TNFα, and TGFβ, and increasing the expression of antioxidant enzymes (89–95). In addition, Fernandes and colleagues found that DHA-enriched fish oil, compared to EPA-enriched fish oil, was better at attenuating renal disease and increasing the survival of NZB/W F1 mice (90). This suggests that the relative abundance of EPA and DHA in fish oil might impact the outcomes of experiments designed to examine the effects of fish oil on SLE. Moreover, a recent study reported that omega-6 PUFAs did not have the same beneficial effect on lupus nephritis as omega-3 PUFAs (96). The disease-ameliorating effect of omega-3 PUFA against murine lupus was further confirmed in several lupus-prone mouse models other than NZB/W F1 (86, 97, 98). Starting late 1980s, more than 10 interventional studies with omega-3 PUFAs as treatments have been done in patients with SLE. Some studies showed promising results, especially for SLE patients with cardiovascular disease, which has emerged as an important cause of death in patients with SLE (99, 100).

While VD, VA, and PUFAs are known to change the composition of gut microtioba (21, 101, 102), how different dietary components modulate the microbiota of SLE patients and subsequent disease is unclear. One recent study has described diet-mediated increases of specific microbial genera that are known to be lower in SLE (103). Further studies are necessary to determine whether the modulation of diet – likely to be less expensive and safer than immunosuppressive drugs – can be effective at establishing a healthy balance between the host and symbiotic microbiota in the gut of SLE patients. If so, diet modulation might become a cost-effective approach for the management of SLE.

## Bacterial Antigens and SLE

Bacteria constitute a large part of the symbiotic microbiota living in our body. Diverse components of Gram-positive and Gram-negative bacteria have been reported to contribute to the initiation and maintenance of lupus disease through stimulating TLRs, especially TLR2 and TLR4. TLRs are pattern recognition receptors that can recognize invading microorganisms bearing pathogen-associated molecular patterns (104). Details of TLR signaling pathways and their effects on autoimmune diseases, including SLE, have been reviewed elsewhere (105). In the current review, we will focus on the roles of bacterial antigens in lupus and their possible link to the sex bias observed in SLE. We hypothesize that commensal bacteria naturally present in our microbiota might provide autoantigens that mediate the development of SLE.

### Lipopolysaccharide

Lipopolysaccharide (LPS) is a Gram-negative cell wall component that can be recognized by TLR4. In SLE patients, soluble CD14 (sCD14), which is released by monocytes in response to LPS, is increased in the blood (106). The level of sCD14 is highly correlated with disease activity parameters, suggesting the involvement of LPS in lupus development. In addition, repeated injections of LPS into lupus-prone mice resulted in increased autoantibody production and development of glomerulonephritis (107–111). Activation of TLR4 also promotes lupus disease activity in transgenic mice (107, 112, 113). Lupus spontaneously develops in mice with overexpression of a molecular chaperone of TLR4 that increases its responsiveness; but when commensal bacterial flora was deleted through treatment with antibiotics, the enhanced lupus phenotype was largely ameliorated (107). This suggests that TLR4 hyperresponsiveness to gut flora (which contains LPS) plays an essential role in lupus development. Moreover, Ni and colleagues found increased levels of serum autoantibodies and more severe lung injury when challenging apolipoprotein E-deficient (ApoE^−/−^) mice with LPS (114). Furthermore, immunization of non-autoimmune mice (C57BL/6 or BALB/c) with phospholipid-binding proteins induced lupus-like disease, and this was facilitated by the presence of LPS (115–117). Taken together, these data suggest that enhanced TLR4 signaling by LPS stimulation is sufficient to induce SLE. LPS might do so by inducing neutrophil activation and migration (118–120), key processes that promote the development of SLE (72). Inhibition of TLR4, on the other hand, reduces autoantibody production and decreases glomerular IgG deposits in the kidney for some lupus-prone murine models (121, 122). However, in TLR4-knockout MRL/lpr mice, disease activity was not modified (123). This may be due to the different genetic backgrounds of the mice strains. Further testing of disease outcome through TLR4 knockout should be done in additional strains of lupus-prone mice to determine the role of TLR4 deficiency in lupus.

In addition to the effect of LPS on neutrophil activation (118–120), several recent studies have explored the mechanisms by which LPS induces lupus. Qin et al. reported that the interaction of TLR4 and LPS strongly induced CD40 expression in macrophages and microglia (124). It was also found that LPS had the ability to increase CD40 mRNA expression in various tissues, including liver and kidney, in NZB/W F1 mice (125). CD40 silencing reduced the glomerular deposits of IgG and C3 in these mice, revealing a possible role of LPS-TLR4-CD40 signaling in the pathogenesis of lupus. Another possible role for LPS-TLR4 in lupus is to induce autoantibody production or isotype switching toward more pathogenic immunoglobulins, like IgG (126). Both MyD88- and TRIF-mediated signaling pathways are believed to contribute to increased autoantibody levels, though TRIF may play a more important role in driving autoantigen-specific IgG response (126). Moreover, it has been found that IL-18 is induced by LPS stimulation and this cytokine may cooperate with LPS–TLR4 in breaking the tolerance in mice with lupus nephritis (127).

Systemic lupus erythematosus is a female-biased disorder. Accumulating evidences have linked TLR4 function to estrogen and estrogen receptor α (ERα). Studies by Gilkeson’s group have found that female SLE patients possess more active monocytes with enhanced TLR4 responsiveness than male SLE patients (128). In lupus-prone mice, ERα deficiency ameliorated renal damage and prolonged survival compared to ERα-sufficient controls (129). Importantly, knocking out ERα in both lupus-prone and control mice resulted in impaired TLR4 activation in immune cells, indicating that estrogen and ER signaling can influence TLR4 responsiveness (130, 131). These results suggest possible contribution of TLR4 activation to sex bias in SLE.

### Other Bacterial Antigens

Lipoteichoic acid (LTA), a major component of Gram-positive bacterial wall, is also reported to be involved in lupus pathogenesis. LTA is a ligand for TLR2, whose expression is increased in T cells, B cells, and monocytes from SLE patients (132). Increased TLR2 leads to enhanced IL-17A and IL-17F production and is associated with inflammatory response of CD4^+^ T cells. In mice, TLR2 activation is known to trigger lupus nephritis (133). In both B6/lpr mice and pristine-induced lupus mice, TLR2 knockout resulted in decreased autoantibody levels and ameliorated lupus-like symptoms (121, 134, 135). However, like the deficiency of TLR4, in MRL/lpr mice, TLR2 deficiency did not affect lupus pathogenesis (123, 136), possibly due to mouse strain differences.

Another bacterial antigen and component of bacterial biofilms, amyloid fiber (curli), has been reported to induce autoantibody production (137). Amyloid fibers can tightly bind to extracellular DNA that exists in many bacterial biofilms. Amyloid-DNA composites have been found to be strong stimulators of both innate and adaptive responses, with the ability to promote IL-6 and TNFα production and type I interferon response in NZB/W F1 mice (138). Importantly, injection of curli-DNA composites greatly increased the autoantibody level in lupus-prone mice, and even stimulated autoantibody production in wild-type mice. Using an amyloid-induced lupus model, Cao and colleagues have recently uncovered important roles of natural killer cells and IFNγ in SLE pathogenesis downstream of type I interferon response (139).

## The “SLE Microbiota”

**Figure 1:**
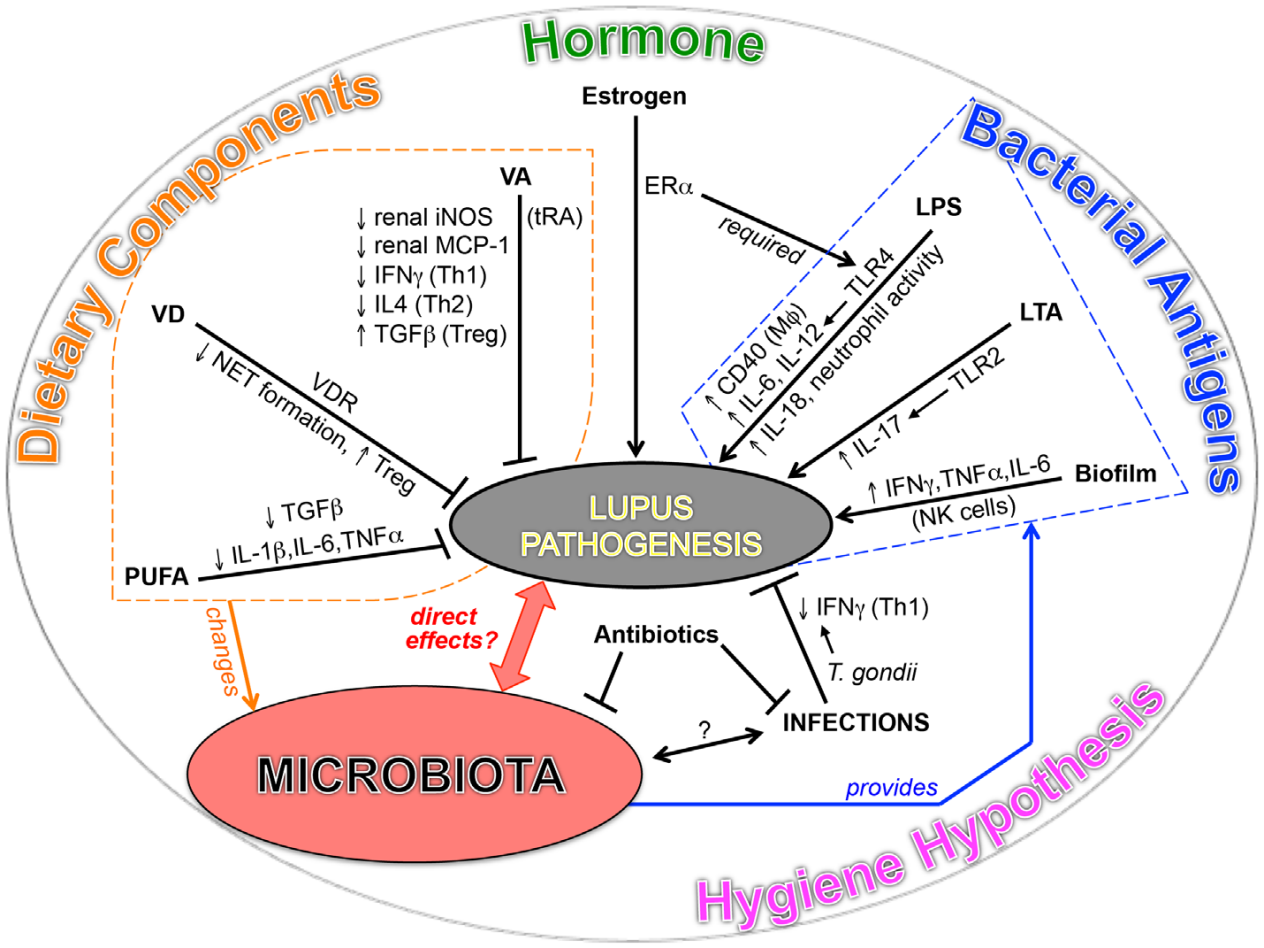
**Emerging evidences point to a potential link between SLE and microbiota**.

The significance of symbiotic microbiota in the development of T1D has been shown in non-obese diabetic mice, which spontaneously develop T1D with a bias toward females (11, 12). The function of microbiota in T1D is found to be highly associated with sex hormones. Fecal transplant of male gut microbiota to female mice ameliorated the disease and increased testosterone. For SLE, although the initial comparison between lupus-prone mice in germ-free vs. conventional housing conditions showed no difference in disease severity (140), emerging evidences in both SLE patients and lupus-prone mice point to a potential link between lupus and microbiota (Figure 1).

Intestinal dysbiosis has been reported in SLE patients. Compared to age- and sex-matched healthy controls, the fecal *Firmicutes/Bacteroidetes* ratio was found to be significantly lower in SLE patients even during remission (22). The same research group also described alterations in the composition and metabolic functions of gut microbiota in SLE (63). In mice, a recent study has shown that ANA production, a hallmark feature of autoimmune diseases that include SLE, is affected by neonatal colonization of gut microbiota (141). Using mice deficient of lymphotoxin-β receptor (LTβR) – the signaling of which controls development of secondary lymphoid organs – the authors found that LTβR-expressing RORγT^+^ innate lymphoid cells, located in the intestinal lamina propria, were important for the maintenance of immunological tolerance. Importantly, it was found that antibiotics-mediated removal of segmented filamentous bacteria (SFB) inhibited the development of ANA (141). However, in another recent study, SFB were found to be unassociated with the outcome of lupus in (SWR × NZB)-F1 (SNF1) mice (23). When given acidic pH water, SNF1 mice showed slower development of nephritis and a lower level of circulating ANA, and the improved outcome was associated with changes of gut microbiota unrelated with SFB (23). In their study, the relative abundance of *Lactobacillus* and the ratio of *Firmicutes/Bacteroidetes* were higher in mice with lower lupus severity (23). These changes were consistent, respectively, with our results in MRL/lpr mice (21) and the findings of microbiota composition in human SLE patients (22). The same authors have also reported the role of gut immune cells in female-biased development of lupus in SNF1 mice (142). Compared to male counterparts, the gut mucosa of female SNF1 mice has a higher frequency of gut-imprinted α4β7 T cells, higher expression of type I interferons, and a larger number of cells secreting IL-17, IL-22, and IL-9 (142). Altogether, the intestinal microenvironment, including microbiota, immune cells and cytokines, could contribute to the development of lupus.

Our research group has recently found that, in female lupus-prone mice, there are significant reduction of *Lactobacillaceae* and increase of *Lachnospiraceae* both prior to disease onset and in the late stage of disease with severe lupus symptoms (21). We also found that lupus-like symptoms, including nephritis, were improved with oral treatment of tRA. Importantly, the improvement was highly associated with the ability of tRA to restore *Lactobacilli* (21). Our work shows the potential benefits of modulating gut microbiota, especially by increasing the level of *Lactobacilli*, in the treatment of lupus. *Lactobacilli* can be introduced as probiotics, which are known to be beneficial to the host when administered in adequate amounts. Proposed health benefits provided by the consumption of *Lactobacilli* include prevention of constipation, hepatic disease, infections, allergies, and as recently suggested, inhibition of autoimmune diseases such as IBD and T1D (143–149). Some *Lactobacillus* strains have been demonstrated to exert specific effects that include modulation of host microbiota, inhibiting the formation of NETs, improving antioxidant status, or increasing the expression of genes encoding junction and adhesion proteins (150–152). This suggests an attractive prospective of utilizing certain strains of *Lactobacillus* in disease management for SLE.

To directly examine the potential effects of sex and gut microbiota on SLE, one approach would be to correct the imbalanced microbial composition associated with SLE with fecal transplantation – from healthy individuals to patients, or from males to females – and see if the correction ameliorates disease symptoms. This is yet to be reported for either lupus-prone mouse models or SLE patients, and remains an area that researchers actively explore.

## Conflict of Interest Statement

The authors declare that the research was conducted in the absence of any commercial or financial relationships that could be construed as a potential conflict of interest.
